# Personality Pathology and Functional Outcomes During Pharmacological Treatment of Adult ADHD

**DOI:** 10.1002/pmh.70071

**Published:** 2026-03-29

**Authors:** Peter Jacobsson, Elin Palage, Christopher J. Hopwood, Bo Söderpalm, Robert F. Krueger, Viktor Tasselius, Thomas Nilsson

**Affiliations:** ^1^ Department of Psychiatry and Neurochemistry, Institute of Neuroscience and Physiology, the Sahlgrenska Academy University of Gothenburg Sweden; ^2^ Psychiatry Halland, Region Halland Sweden; ^3^ University of Zurich Zurich Switzerland; ^4^ Department of Psychology University of Minnesota Minneapolis Minnesota USA; ^5^ School of Public Health and Community Medicine, Institute of Medicine, the Sahlgrenska Academy University of Gothenburg Sweden; ^6^ Centre for Ethics, Law and Mental Health (CELAM), Institute of Neuroscience and Physiology University of Gothenburg Gothenburg Sweden; ^7^ Forensic Psychiatry Sahlgrenska University Hospital Gothenburg Sweden

**Keywords:** adult ADHD, AMPD, dimensional personality pathology, functional impairment, ICD‐11, pharmacology, stimulant treatment, treatment response

## Abstract

Adults with ADHD show substantial variability in response to pharmacological treatment, and comorbid personality pathology may help explain why symptom improvement often fails to translate into meaningful functional improvement. This naturalistic longitudinal study examined whether dimensional personality pathology predicts symptomatic and functional outcomes during routine stimulant treatment. A total of 246 adults with ADHD (66% women; mean age = 33.5) contributed repeated assessments over irregular follow‐up intervals, with substantial attrition across waves. Linear mixed‐effects models were used to accommodate unbalanced repeated measures. Linear mixed‐effects models indicated that higher personality dysfunction was strongly associated with less improvement in functioning (*β* = 0.44, *p* < 0.001) and more persistent ADHD symptoms (*β* = 0.20, *p* < 0.001), independent of stimulant dose and time in treatment. Negative affectivity, detachment, psychoticism, and disinhibition were the maladaptive trait domains most consistently linked to poorer functional outcomes, whereas disinhibition showed the strongest association with residual ADHD symptom burden. Medication‐related effects were modest in comparison. These findings identify personality pathology as a clinically relevant source of heterogeneity in adult ADHD treatment response and support routine assessment of personality functioning to inform clinical decision‐making.

## 1 Introduction

Adult ADHD is a neurodevelopmental disorder affecting approximately 2%–5% of adults (Ayano et al. 2023; Song et al. 2021). It is characterized by inattention, hyperactivity, and impulsivity, often accompanied by emotional dysregulation, and diagnosis requires evidence of clinically significant functional impairment across multiple domains (APA 2013; Barkley 2018; Hirsch et al. 2019; Kosheleff et al. 2023; WHO 2004).

Pharmacological treatment—typically stimulant medication—is first‐line and effective for reducing ADHD symptoms in adults, with non‐stimulants recommended as second‐line options (Chan et al. 2020; Wilens et al. 2011). In contrast, effects on functional outcomes are more variable, and most trials emphasize symptom reduction rather than meaningful functional improvement (Coghill et al. 2017; Kosheleff et al. 2023).

Psychiatric comorbidity, including personality pathology, is highly prevalent in adults with ADHD (Katzman et al. 2017). Symptom overlap with other psychiatric conditions has contributed to dimensional frameworks that conceptualize psychopathology along continua rather than discrete categories (Forbes et al. 2024; Kotov et al. 2017; Krueger and Markon 2014). This approach is formalized in the DSM‐5 Alternative Model of Personality Disorder (AMPD; APA 2013) and the ICD‐11 personality disorder model (WHO 2022) which assess general personality dysfunction alongside maladaptive trait domains. These dimensions are particularly relevant to adult ADHD given shared features such as impulsivity, emotional dysregulation, attentional instability, and interpersonal difficulties. Within this framework, the Level of Personality Functioning Scale (LPFS) indexes self and interpersonal dysfunction, while the Personality Inventory for DSM‐5 (PID‐5) captures maladaptive trait expressions.

Consistent with this perspective, prior studies have shown strong associations between ADHD and maladaptive personality traits, particularly disinhibition, which overlaps closely with core ADHD symptoms (Gomez and Corr 2014; Jacobsson et al. 2024; Smith and Samuel 2017). Our earlier work further identified distractibility—a lower order disinhibition facet—as especially prominent in adults with ADHD. It demonstrated that both personality dysfunction and maladaptive traits are associated with pharmacological treatment processes (Jacobsson et al. 2025).

Despite substantial symptom overlap between adult ADHD and dimensional models of personality pathology, the dominant treatment paradigms have evolved in strikingly different directions. ADHD continues to be conceptualized primarily as a neurodevelopmental condition, with treatment emphasizing pharmacological symptom reduction. In contrast, personality pathology is understood as involving enduring disturbances in self and interpersonal functioning, addressed through psychotherapeutic approaches. These divergent traditions may obscure opportunities for integrated, mechanism‐based treatments that address shared features across both conditions (Haijen et al. 2024; Ostinelli et al. 2025).

Whereas prior studies have largely focused on medication efficacy or predictors of treatment adherence and discontinuation, less is known about how enduring personality pathology shapes functional outcomes during pharmacological treatment in adults with ADHD. The present study addresses this gap by testing whether personality pathology, conceptualized within the AMPD framework, moderates functional and symptomatic improvement during pharmacological treatment in adults with ADHD.

We focused on general personality dysfunction, as indexed by the LPFS‐BF 2.0, as a primary predictor given its role as an indicator of overall personality severity within dimensional models of personality disorder (AMPD; ICD‐11). In addition, we examined disinhibition as a theoretically salient trait domain given its conceptual overlap with core ADHD features such as impulsivity, distractibility, and behavioral dysregulation. Both functional impairment and ADHD symptom levels were modeled as outcomes, as pharmacological treatment primarily targets symptom reduction, whereas broader adaptive functioning may depend more strongly on enduring personality‐related capacities.

## 2 Methods

### 2.1 Participants and Procedure

Data from this clinical cohort have partly been used in a previously published study examining predictors of premature discontinuation of pharmacological treatment in adult ADHD (Jacobsson et al. 2025). The present study addresses a distinct research question by focusing on *functional and symptomatic outcomes during ongoing treatment* and by examining dimensional personality pathology as a moderator of treatment‐related improvement.

Sampling took place between 2018 and spring 2025. Diagnostic evaluations followed local protocols and included (a) a clinical interview covering developmental and current symptoms, supplemented when possible by collateral informants, and (b) a standardized set of self‐report measures assessing ADHD symptoms, functional impairment, general psychiatric concerns, and personality functioning (descriptive statistics for all baseline variables are provided in Table S1). Diagnostic evaluations included a structured clinical assessment of DSM‐5 ADHD criteria, confirmation of childhood‐onset symptoms, and evaluation of functional impairment across domains. Clinicians routinely assessed differential diagnoses, including trauma‐related disorders, mood disorders, and substance use disorders, to minimize diagnostic misclassification.

Follow‐up intervals ranged from 7 to 2318 days (median = 735; IQR = 383–1127; see Table S2), reflecting the naturalistic scheduling of routine psychiatric care rather than fixed research waves. The COVID‐19 pandemic further contributed to extended intervals.

At the first follow‐up measurement point (T1), 217 participants provided World Health Organization Disability Assessment Schedule (WHODAS) data, and 165 provided ADHD symptom data, while fewer than 10 participants contributed data at T5 and T6 (see Table 1).

**TABLE 1:** Participant retention across follow‐up waves (WHODAS 2.0 and ADHD symptoms).

Wave	Visit (T)	WHODAS 2.0 (*N*)	ADHD (*N*)
1	T1	217	165
2	T2	79	69
3	T3	23	20
4	T4	10	9
5	T5	3	2
6	T6	2	2

*Note:* Retention decreased across waves for both outcomes. In absolute terms, the largest drop occurred between T1 and T2, while in relative terms, the steepest proportional decline was between T2 and T3 (only about 29% of participants remained).

The total sample included 246 adults with ADHD (66% women; M age = 33.5, SD = 9.9). Overlapping participation yielded analytic subsamples of 184–235 individuals with at least one repeated observation. Given this heterogeneity, the study design is best described as a naturalistic longitudinal cohort with unbalanced repeated measures.

### 2.2 Medication Use

Relevant clinical data were manually extracted from patient progress notes. All participants were prescribed pharmacological treatment before the initial repeated‐measure point. Data extraction was conducted by a psychiatry resident (E.P.) who was blinded to personality data, while treating clinicians were unaware of the personality assessment results. Extracted variables included the *date of treatment initiation*, *medication type and dosage*, and any *continuation, discontinuation, or change within 30 days* of each self‐report assessment (see Supporting Information S3 for more detailed information).

Amphetamine‐equivalent dosages were calculated using standard conversion ratios (see Supporting Information S4). Because detailed titration data were unavailable, *treatment duration* served as the primary proxy for cumulative exposure.

### 2.3 Measures

#### 2.3.1 WHODAS 2.0—12‐Item Version

The 12‐item WHODAS 2.0 (Üstün, 2010) is a standardized measure of functioning across six life domains based on the ICF framework. Respondents rate difficulty performing daily activities over the past 30 days on a 5‐point Likert scale (1 = *none* to 5 = *extreme or cannot do*). Internal consistency in this study was *α* = 0.88.

#### 2.3.2 Level of Personality Functioning Scale—Brief Form 2.0 (LPFS‐BF 2.0)

The LPFS‐BF 2.0 (Weekers et al. 2019) assesses general personality dysfunction across self and interpersonal functioning. It consists of 12 items rated on a 4‐point Likert scale. Internal consistency was *α* = 0.86.

#### 2.3.3 PID‐5

The PID‐5 (APA 2013; Krueger et al. 2012) measures maladaptive personality traits across five higher order domains and 25 lower order facets. Domain scores were calculated using the APA‐recommended 15‐facet subset (three per domain). Internal consistency at the domain level ranged from *α* = 0.85 to 0.94 and from *α* = 0.36 to 0.88 at the facet level (see Table S5 for all domains and facets).

#### 2.3.4 ADHD Symptom Measures

Two self‐report measures of adult ADHD symptoms were used: the Adult ADHD Self‐Report Scale (ASRS v1.1; Kessler et al. 2005) and the Barkley Current Symptoms Scale (CSS; Barkley 2006). Each includes 18 items corresponding to DSM symptom criteria, rated on 5‐ and 4‐point Likert scales, respectively. During data collection, the regional protocol transitioned from the CSS to the ASRS. Scores from both scales were standardized and combined into a unified ADHD symptom score. Internal consistency for the unified score was *α* = 0.91.

#### 2.3.5 Statistical Analyses

All analyses were conducted using R version 4.4.0 (Team 2024). Preliminary descriptive analyses examined age and sex differences in personality dysfunction, maladaptive traits, functional impairment, and ADHD symptom burden using independent‐samples *t*‐tests and Pearson correlations (see Tables S6 and S7). Older age was associated with lower personality pathology and slightly lower functional impairment, while women showed higher personality dysfunction, Negative Affectivity, and functional impairment. Age and sex were therefore included as covariates in all subsequent models; their inclusion did not materially alter effect estimates, and no significant sex‐by‐personality interactions were observed.

Because detailed titration data were unavailable, treatment duration was used as the primary proxy for cumulative medication exposure, with amphetamine‐equivalent dose at each assessment included as a secondary covariate. Personality variables were included as predictors of longitudinal functional and symptomatic outcomes, rather than as predictors of treatment discontinuation. Given the unbalanced longitudinal design with irregular follow‐up intervals, linear mixed‐effects models (LMMs) were used to analyze repeated measures. This approach accommodates missing data and within‐subject dependence under the missing‐at‐random assumption. All models included random intercepts for participants; random slopes for time were tested but did not improve model fit and were excluded. Because repeated outcome measures (including baseline) were modeled simultaneously, baseline levels were incorporated into the longitudinal trajectory estimation.

Two model sets were specified: (a) functional‐impairment models with WHODAS 2.0 scores as the outcome, and (b) ADHD‐symptom models with z‐standardized ASRS/CSS composite scores as the outcome. Predictors were z‐standardized and included treatment duration, amphetamine‐equivalent dose, general personality dysfunction (LPFS‐BF 2.0), and maladaptive personality traits (PID‐5 domains or facets).

Models were estimated using restricted maximum likelihood in *lme4* (Bates et al. 2015), with Satterthwaite‐adjusted degrees of freedom from *lmerTest* (Kuznetsova et al. 2017). Nested models were compared using likelihood‐ratio tests, and model diagnostics assessed residual distributions, multicollinearity, and influential observations. Marginal and conditional R^2^ values were calculated using *MuMIn* (Bartoń 2015). Main‐effects models were retained, as interaction terms did not improve model fit (ΔAIC < 3; ΔBIC < 5) and yielded unstable estimates. Positive standardized coefficients (*β*) indicate poorer outcomes (i.e., less improvement) over time.

#### 2.3.6 Use of AI‐Assisted Tools

AI‐based tools were used for marginal language refinement and limited programming assistance (e.g., refinement of R scripts) during manuscript preparation. All analyses, interpretations, and conclusions are the author's own, and the author takes full responsibility for the content of this manuscript.

## 3 Results

### 3.1 Model Fit and Predictors

#### 3.1.1 General Personality Dysfunction

Higher levels of general personality dysfunction were associated with greater functional impairment (*β* = 0.44, *p* < 0.001) and higher ADHD symptom burden (*β* = 0.20, *p* < 0.001), indicating less improvement over time among participants with elevated personality pathology (Tables 2 and 3). These associations remained robust after adjustment for treatment duration, stimulant dosage, age, and sex.

**TABLE 2:** Personality dysfunction, domain, and facet‐level effects on changes in functional impairment. Linear mixed‐effects models adjusted for stimulant dose and treatment duration.

		*β*	CI	*p*
General personality dysfunction		0.44	[0.35, 0.54]	< 0.001
Negative affectivity		0.37	[0.27, 0.47]	< 0.001
Detachment		0.35	[0.24, 0.46]	< 0.001
Antagonism		0.13	[0.01, 0.24]	0.032
Disinhibition		0.29	[0.18, 0.41]	< 0.001
Psychoticism		0.33	[0.22, 0.43]	< 0.001
Negative affectivity	Emotional lability	0.06	[−0.06, 0.18]	0.306
	Anxiousness	0.40	[0.30, 0.51]	< 0.001
	Separation insecurity	0.26	[0.15, 0.37]	< 0.001
	Submissiveness	0.37	[0.26, 0.47]	< 0.001
	Depressivity	0.28	[0.17, 0.39]	< 0.001
Detachment	Withdrawal	0.36	[0.26, 0.47]	< 0.001
	Anhedonia	0.18	[0.07, 0.30]	0.002
	Intimacy avoidance	0.29	[0.19, 0.40]	< 0.001
	Restricted affectivity	0.18	[0.06, 0.30]	0.003
	Suspiciousness	0.36	[0.26, 0.47]	< 0.001
Antagonism	Manipulativeness	0.09	[−0.03, 0.20]	0.145
	Deceitfulness	0.14	[0.02, 0.25]	0.018
	Grandiosity	0.28	[0.18, 0.39]	< 0.001
	Hostility	0.25	[0.14, 0.37]	< 0.001
	Callousness	0.05	[−0.07, 0.16]	0.437
	Attention seeking	0.11	[−0.01, 0.22]	0.074
Disinhibition	Irresponsibility	0.25	[0.13, 0.36]	< 0.001
	Impulsivity	0.21	[0.09, 0.32]	< 0.001
	Distractibility	0.21	[0.09, 0.32]	< 0.001
	Perseveration	0.24	[0.13, 0.35]	< 0.001
	Rigid perfectionism	0.19	[0.08, 0.30]	< 0.001
	Risk taking	0.19	[0.07, 0.31]	0.002
Psychoticism	Unusual beliefs	0.28	[0.17, 0.39]	< 0.001
	Eccentricity	0.36	[0.26, 0.47]	< 0.001
	Perceptual dysregulation	0.21	[0.09, 0.32]	< 0.001

*Note:* Standardized estimates (*β*) and 95% confidence intervals are reported. Higher WHODAS 2.0 scores indicate greater functional impairment; positive *β* values therefore represent poorer outcomes (i.e., less improvement) over time.

**TABLE 3:** Personality dysfunction, domain and facet level effect on changes in ADHD symptom levels. Linear mixed‐effects models adjusted for stimulant dose and treatment duration.

		*β*	CI	*p*
General personality dysfunction		0.20	[0.10, 0.29]	< 0.001
Negative affectivity		0.20	[0.08, 0.31]	< 0.001
Detachment		0.07	[−0.05, 0.18]	0.244
Antagonism		0.17	[0.06, 0.28]	0.003
Disinhibition		0.35	[0.24, 0.46]	< 0.001
Psychoticism		0.21	[0.10, 0.32]	< 0.001
Negative affectivity	Submissiveness	0.04	[−0.07, 0.16]	0.453
	Depressivity	0.11	[−0.01, 0.22]	0.066
	Separation insecurity	0.18	[0.06, 0.29]	0.002
	Anxiousness	0.10	[−0.01, 0.22]	0.077
	Emotional lability	0.20	[0.09, 0.31]	< 0.001
Detachment	Suspiciousness	0.24	[0.13, 0.35]	< 0.001
	Restricted affectivity	0.07	[−0.05, 0.18]	0.241
	Withdrawal	0.02	[−0.09, 0.14]	0.680
	Intimacy avoidance	0.05	[−0.07, 0.17]	0.383
	Anhedonia	0.11	[−0.01, 0.22]	0.065
Antagonism	Manipulativeness	0.12	[0.01, 0.24]	0.029
	Deceitfulness	0.15	[0.03, 0.26]	0.010
	Hostility	0.18	[0.07, 0.29]	0.002
	Callousness	0.13	[0.01, 0.24]	0.028
	Attention seeking	0.15	[0.03, 0.26]	0.012
	Grandiosity	0.17	[0.06, 0.29]	0.003
Disinhibition	Irresponsibility	0.24	[0.13, 0.35]	< 0.001
	Impulsivity	0.26	[0.15, 0.37]	< 0.001
	Distractibility	0.29	[0.18, 0.40]	< 0.001
	Perseveration	0.11	[−0.01, 0.22]	0.064
	Rigid perfectionism	0.08	[−0.03, 0.20]	0.138
	Risk‐taking	0.07	[−0.05, 0.19]	0.227
Psychoticism	Eccentricity	0.22	[0.10, 0.33]	< 0.001
	Perceptual dysregulation	0.16	[0.05, 0.27]	0.005
	Unusual beliefs	0.16	[0.05, 0.28]	0.004

*Note:* Standardized estimates (*β*) and 95% confidence intervals are reported. Higher ASRS v1.1/CSS scores indicate higher ADHD symptom levels; positive *β* values therefore represent poorer outcomes (i.e., less improvement) over time.

#### 3.1.2 Maladaptive Personality Domains and Facets

**FIGURE 1:**
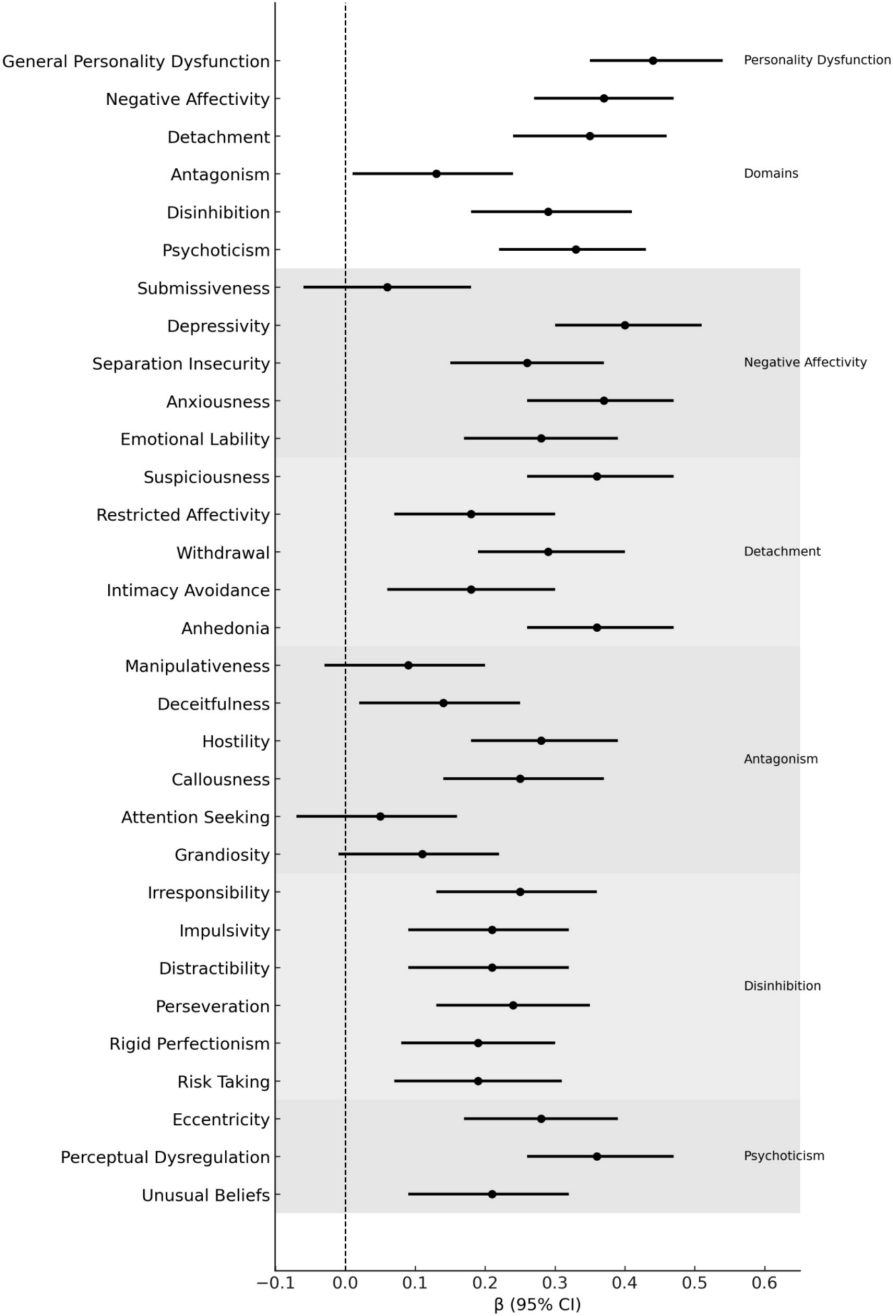
Changes in functional impairment related to personality pathology during medication in adult ADHD. *Note.* Standardized regression coefficients (*β*) with 95% confidence intervals derived from linear mixed‐effects models predicting change in functional impairment (WHODAS 2.0) from personality dysfunction (LPFS‐BF 2.0) and maladaptive personality traits (PID‐5 domains and facets) predicting change in functional impairment (WHODAS 2.0). Positive *β* values represent less improvement (i.e., greater persistence of impairment) with higher levels of the personality variable; negative *β* values represent greater improvement. Shaded bands indicate domain groupings; the dashed line marks *β* = 0.

Across domain‐level models, Negative Affectivity (*β* = 0.37, *p* < 0.001), Detachment (*β* = 0.35, *p* < 0.001), Psychoticism (*β* = 0.33, *p* < 0.001), and Disinhibition (*β* = 0.29, *p* < 0.001) were most strongly associated with poorer *functional* outcomes, reflecting limiting improvements in everyday functioning during pharmacological treatment (Table 2; Figure 1). At the facet level, anxiousness, suspiciousness, submissiveness, and depressivity showed relatively larger effects, with smaller but consistent contributions from Anhedonia and Perceptual Dysregulation.

**FIGURE 2:**
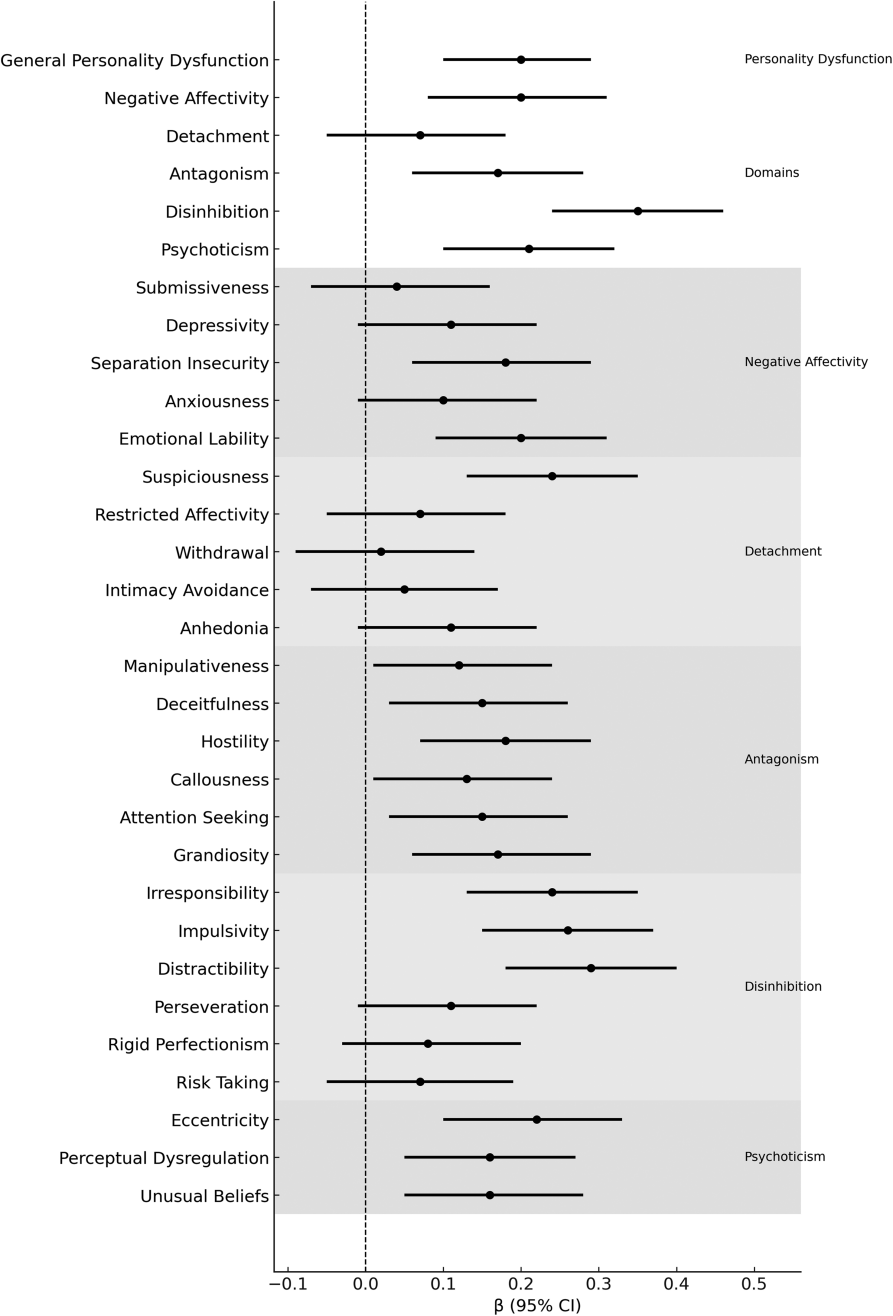
Changes in ADHD symptoms related to personality pathology during medication in adult ADHD. *Note.* Standardized regression coefficients (*β*) with 95% confidence intervals derived from linear mixed‐effects models predicting changes in ADHD symptom levels (ASRS v1.1/CSS) from personality dysfunction (LPFS‐BF 2.0) and maladaptive personality traits (PID‐5 domains and facets), controlling for stimulant dose and treatment duration. Positive *β* values represent less symptom reduction (i.e., smaller improvement); negative *β* values represent greater improvement. Shaded bands indicate domain groupings; the dashed line marks *β* = 0.

Disinhibition emerged as the domain most strongly associated with persistent ADHD *symptoms* (*β* = 0.35, *p* < 0.001), with Irresponsibility, Impulsivity, and Distractibility showing the largest facet‐level effects (Table 3; Figure 2). Smaller but reliable associations were also observed for Negative Affectivity, Psychoticism, and selected Antagonism facets, suggesting that affective instability and interpersonal antagonism modestly contributed to residual symptom burden.

### 3.2 Treatment Duration and Stimulant Dosage

Because detailed titration data were unavailable, treatment duration was interpreted as a proxy for cumulative medication exposure. Time in treatment showed weak associations with outcome, reaching nominal significance for functional impairment (*β* = −0.11, *p* = 0.05) and a non‐significant trend for ADHD symptoms. In contrast, amphetamine‐equivalent dosage showed small but statistically reliable associations with improvement in both outcomes (*β* = −0.08 to −0.10, *p* < 0.002). Overall, medication‐related predictors accounted for limited variance relative to personality pathology.

### 3.3 Model Comparisons and Overall Change

Additive main‐effects models consistently outperformed interaction models (ΔAIC < 3; ΔBIC < 5). Interaction terms between personality variables and treatment duration or dosage did not improve model fit and yielded unstable estimates, likely due to sparse follow‐up data.

Across the observation period, functional impairment declined significantly (mean reduction = 0.26 points, 95% CI [0.16, 0.36], *t*(188) = 5.14, *p* < 0.001), and ADHD symptoms decreased by an average of 0.26 z‐units (*t*(224) = 6.26, *p* < 0.001). Both outcomes showed moderate temporal stability (functional impairment: ρ = 0.37; ADHD symptoms: ρ = 0.36; both *p* < 0.001). All models were adjusted for age and sex, and no sex‐by‐personality interactions were detected. Model fit indices indicated meaningful variance explained by the fixed predictors. The LPFS model predicting functional impairment yielded a marginal R^2^ of 0.23 and a conditional R^2^ of 0.48, indicating that fixed effects accounted for approximately 23% of total variance in functional outcomes, while the full model (including random intercept variance) accounted for 48%. The corresponding LPFS model predicting ADHD symptoms yielded a marginal R^2^ of 0.11 (conditional R^2^ = 0.48). When Disinhibition was examined as the primary predictor of ADHD symptoms, the marginal R^2^ increased to 0.18, indicating meaningful explanatory capacity beyond medication and demographic covariates.

### 3.4 Attrition Analyses

Baseline differences between participants retained to ≥T2 and early dropouts (≤T1 or baseline‐only) are presented in Table 4. No significant differences were observed in age, functional impairment (WHODAS 2.0), overall personality dysfunction (LPFS‐BF 2.0), or any PID‐5 domain scores (all *p* > 0.11). Effect sizes were uniformly small (|d| ≤ 0.19). Sex distribution also did not differ significantly between groups (*p* = 0.15, Cramér's V = 0.08). These findings suggest that attrition was not systematically related to baseline demographic or clinical characteristics.

**TABLE 4:** Baseline differences between participants retained to ≥ T2 and early dropouts.

	T2+ (*n* = 93)	Early dropout (*n* = 236)	*p*	Effect size
Age	32.98	33.19	0.863	*d* = −0.02
Sex (female %)	69.9%	60.6%	0.148	*V* = 0.08
WHODAS 2.0	1.60	1.48	0.119	*d* = 0.19
LPFS‐BF 2.0	1.54	1.59	0.533	*d* = −0.08
Negative affectivity	1.40	1.51	0.179	*d* = −0.18
Detachment	1.17	1.18	0.920	*d* = −0.01
Antagonism	0.78	0.82	0.593	*d* = −0.07
Disinhibition	1.66	1.64	0.740	*d* = 0.04
Psychoticism	0.85	0.94	0.214	*d* = −0.17

*Note:* No comparisons remained significant after false discovery rate correction. Effect sizes were small across all variables.

## 4 Discussion

This study examined why functional outcomes vary among adults with ADHD receiving pharmacological treatment, focusing on the role of dimensional personality pathology. Unlike prior analyses from this cohort that focused on premature treatment discontinuation, the present findings highlight how personality pathology constrains functional gains among individuals who remain in pharmacological treatment. Routine assessment of personality functioning may help identify adults with ADHD who are unlikely to achieve meaningful functional gains from stimulant medication alone and who may *benefit from earlier integration of psychosocial or skills‐based interventions*. The personality dimensions represent complementary aspects of personality pathology: the LPFS‐BF 2.0 indexes the severity of self‐ and interpersonal dysfunction, whereas the PID‐5 describes stylistic trait expressions that shape how these difficulties manifest in daily life.

Higher levels of both general dysfunction and maladaptive traits were associated with slower improvement in functioning and symptoms during treatment, underscoring their role as limiting factors in medication response. These findings emphasize the need for treatment models that integrate pharmacological and personality‐focused interventions to address both symptomatic and functional dimensions of adult ADHD.

These findings primarily reflect the effects of pharmacotherapy as delivered in routine psychiatric care and indicate that, under naturalistic conditions where medication is the primary intervention, personality pathology substantially constrains meaningful functional improvement.

Stimulant medication effectively reduces core ADHD symptoms but often yields limited gains in adaptive functioning; personality‐related impairments appear to restrict how symptom relief translates into everyday improvement. This pattern aligns with prior evidence that stimulant medication primarily targets attentional and behavioral dysregulation (Shaw et al. 2014), whereas personality disturbance reflects more pervasive difficulties in emotion regulation, goal‐directed behavior, and interpersonal relatedness that influence adaptive capacity (Nazari et al. 2025).

General personality dysfunction emerged as the strongest factor limiting improvement in functioning, outweighing the influence of stimulant dose and treatment duration. In other words, higher levels of personality dysfunction were associated with smaller functional gains during pharmacological treatment. Maladaptive personality traits further specified this association by identifying particular affective, interpersonal, and disinhibitory styles that were most strongly linked to poorer functional outcomes.

The observed pattern of associations partly reflects conceptual alignment among the instruments used. The LPFS‐BF 2.0 assesses self‐ and interpersonal functioning, while the PID‐5 captures trait tendencies that shape these capacities in daily life; together, they map closely onto the adaptive domains assessed by the WHODAS 2.0. Because the WHODAS integrates both executive (e.g., attention, work performance) and broader relational or participatory domains, its total score reflects a composite of cognitive‐behavioral efficiency and psychosocial adaptation. This alignment helps explain why personality pathology showed stronger and more consistent associations with functional improvement than with ADHD symptom change, which reflects narrower regulatory processes more directly targeted by medication.

This distinction helps explain the partial dissociation between symptom relief and functional gains observed in the present study. Pharmacotherapy primarily improves attentional and behavioral regulation, whereas broader improvements in everyday functioning depend on emotional stability, motivation, and interpersonal effectiveness—capacities rooted in personality functioning and associated maladaptive personality expressions. Therefore, individuals with higher levels of personality pathology showed smaller functional gains despite symptom improvement, underscoring the need for interventions beyond medication to address enduring self‐ and interpersonal difficulties.

Symptom relief and functional improvement also diverged in their underlying trait patterns. Disinhibition was linked to both ADHD symptom and functional outcomes, indicating a shared regulatory vulnerability, whereas traits reflecting internalizing or relational dysfunction showed stronger associations with everyday adaptation. Disinhibition was uniquely associated with persistent ADHD symptoms, reinforcing the clinical relevance of trait impulsivity for understanding partial stimulant response. In contrast, traits reflecting Negative Affectivity, Detachment, and Psychoticism showed the strongest and most consistent associations with impaired functioning, suggesting that affective instability, social withdrawal, and perceptual or cognitive dysregulation particularly limit adaptive improvement in adults with ADHD. Effects for Antagonism were comparatively small, suggesting that emotional and interpersonal traits may be more relevant to functional outcomes than externalizing tendencies in adults with ADHD.

In this naturalistic sample, stimulant dosage was modestly and consistently associated with symptom improvement but showed no meaningful association with functional change, whereas treatment duration demonstrated weaker and largely non‐significant effects on either outcome. However, attrition and clinical follow‐up patterns likely reflect symptom persistence and treatment engagement rather than exposure per se. As such, the weak associations between treatment duration and outcomes should be interpreted cautiously, particularly given that dosage—though statistically reliable—still accounted for only a small proportion of variance in improvement. These results reinforce the dissociation between symptomatic and functional improvement, underscoring the need to evaluate treatment success using functioning‐focused endpoints rather than symptom counts alone.

Personality pathology remains substantially underrecognized in routine psychiatric care (Tyrer et al. 2015). Only a minority of participants carried a formal personality disorder diagnosis, yet dimensional measures revealed pervasive subthreshold dysfunction that significantly influenced outcomes. These findings demonstrate that personality functioning exerts clinically meaningful effects on adaptation even within populations not meeting categorical PD criteria. Integrating dimensional personality assessment into routine ADHD care could therefore improve prediction of treatment response and guide interventions that target both symptomatic and personality‐related mechanisms. From a clinical perspective, the LPFS‐BF 2.0 is brief (12 items), easy to administer, and demonstrates meaningful prognostic value in this study. Incorporating dimensional personality functioning into standard ADHD assessments may help identify individuals who are less likely to achieve meaningful functional improvement with pharmacotherapy alone and support earlier integration of psychosocial interventions.

More broadly, the findings contribute to an increasingly integrative perspective in which neurodevelopmental symptoms and personality dysfunction are best conceptualized as interacting dimensions rather than discrete diagnostic entities.

## 5 Limitations and Future Directions

Several limitations should be considered. First, attrition over time was substantial, as is common in naturalistic longitudinal clinical studies. However, attrition analyses indicated no significant baseline differences between participants retained to ≥T2 and early dropouts in age, functional impairment, overall personality dysfunction, PID‐5 domains, or sex, and effect sizes were uniformly small. These findings reduce concern regarding systematic attrition bias, although unmeasured factors may still have influenced continued participation.

Although linear mixed‐effects models allowed inclusion of all available observations, non‐random attrition cannot be ruled out. These models assume data are missing at random, an assumption that cannot be directly verified; if missingness was related to unobserved factors such as treatment response or personality pathology, effect estimates may be biased.

Medication exposure was approximated using time in treatment rather than detailed titration or adherence data. As a result, medication effects likely reflect routine clinical practice rather than controlled dosing. In addition, because individuals with more severe or persistent difficulties may remain in treatment longer or receive higher doses, associations between treatment duration, dosage, and outcomes may underestimate true effects or appear reversed. Information on concurrent psychological or skills‐based interventions was not systematically recorded and could not be included in the models. Therefore, we cannot exclude the possibility that non‐pharmacological treatments contributed to functional or symptomatic change.

All measures were self‐reported, raising the possibility of shared‐method variance and underrepresentation of certain traits, particularly antagonistic features. Future studies should incorporate clinician and informant ratings, as well as objective indicators of functioning. Finally, the sample comprised medicated adults from a single Swedish region, which may limit generalizability to non‐medicated populations, other treatment contexts, or younger age groups.

Future research needs to examine how dimensional personality dysfunction interacts with specific psychosocial interventions in shaping functional recovery and symptom trajectories. Ongoing work is aimed at systematically evaluating treatment modalities within this clinical cohort to determine how non‐pharmacological interventions may differentially influence outcomes in individuals with varying levels of personality dysfunction.

## 6 Conclusion

This study demonstrates that while stimulant treatment effectively reduces ADHD symptoms, improvement in broader adaptive functioning is more strongly influenced by underlying personality pathology. Routine assessment of personality pathology may improve prognostic accuracy and support more personalized, multimodal treatment strategies in adult ADHD, particularly for patients showing limited functional improvement despite adequate symptom control. Personality‐informed interventions—whether through tailored psychotherapy or integrated care approaches—may be essential for translating symptomatic improvement into durable, real‐world adaptive gains.

## Funding

The authors have nothing to report.

## Ethics Statement

Ethical approval was granted by the Ethics Review Board in Lund, Sweden (Dnr: 2018/53), in accordance with the Declaration of Helsinki.

## Conflicts of Interest

The authors declare no conflicts of interest.

## Supporting information


**Table S1:** Descriptive statistics for ADHD symptoms, functional impairment, personality dysfunction, maladaptive personality domains, and facets. *Note*. *N* = sample size; M = mean; SD = standard deviation. ADHD symptoms measured with the Current Symptom Scale (CSS) and Adult ADHD Self‐Report Scale (ASRS v.1.1); functional impairment assessed using the World Health Organization Disability Assessment Schedule (WHODAS 2.0); personality dysfunction measured with the Level of Personality Functioning Scale—Brief Form (LPFS‐BF 2.0). Domains and facets assessed using the Personality Inventory for DSM‐5 (PID‐5). ADHD symptoms were converted to *z*‐scores; all other scores are raw scores.

**Table S2:** Follow‐up (time span) and number of measuring points per individual.

**Supporting Information S3:** Descriptives of data extraction of medication.

**Supporting Information S4:** Conversion and rationale amphetamine equivalence.

**Table S5:** Internal consistency of the PID‐5 domains and facets. Notes: Cronbach's *α* and McDonald's *ω* are measures of internal consistency reliability. Values ≥ 0.70 are generally considered acceptable, though interpretation may vary depending on construct complexity. Mean (SD) values represent the average item scores for each domain or facet of the Personality Inventory for DSM‐5 (PID‐5). Higher scores indicate greater expression of the respective trait. Domain‐level indices (in italics) reflect aggregated facet scores.

**Table S6:** Sex differences in personality pathology, functional impairment, and ADHD symptoms (independent‐sample *t*‐tests)*. Note. N* = 246. Independent‐sample *t*‐tests compare male and female participants. *p* < 0.05*, *p < 0.01, **p < 0.001.

**Table S7:** Correlations between age and study variables (Pearson's *r*). *Note. N* = 246. LPFS‐BF 2.0 = Level of Personality Functioning Scale—Brief Form 2.0; PID‐5 = Personality Inventory for DSM‐5; WHODAS 2.0 = World Health Organization Disability Assessment Schedule (12‐item version); ASRS = Adult ADHD Self‐Report Scale v1.1; CSS = Barkley ADHD Current Symptoms Scale. *p* < 0.05*, *p < 0.01, **p < 0.001.

## Data Availability

The data that support the findings of this study are available from the corresponding author upon reasonable request, subject to ethical approval and data protection regulations. All participants consented to participate in this study.
