# Supplementary material for: Personality Pathology and Functional Outcomes During Pharmacological Treatment of Adult ADHD
Source: Personal Ment Health. 2026 Mar 29;20(2):e70071. doi: 10.1002/pmh.70071 (PMC13033909; doi:10.1002/pmh.70071)
Supplement: Supplementary file 1 — Table S1: Descriptive statistics for ADHD symptoms, functional impairment, personality dysfunction, maladaptive personality domains, and facets. Note. N = sample size; M = mean; SD = standard deviation. ADHD symptoms measured with the Current Symptom Scale (CSS) and Adult ADHD Self‐Report Scale (ASRS v.1.1); functional impairment assessed using the World Health Organization Disability Assessment Schedule (WHODAS 2.0); personality dysfunction measured with the Level of Personality Functioning Scale—Brief Form (LPFS‐BF 2.0). Domains and facets assessed using the Personality Inventory for DSM‐5 (PID‐5). ADHD symptoms were converted to z‐scores; all other scores are raw scores. [file PMH-20-0-s007.docx]

**Supplementary Table S1**

*Descriptive Statistics for ADHD Symptoms, Functional Impairment, Personality Dysfunction, Maladaptive Personality Domains, and Facets*

|  | *N* | *M* | *SD* | Min | Max |
| --- | --- | --- | --- | --- | --- |
| **ADHD Symptoms**  (ASRS v1.1/CSS) | 231 | 0.17 | 0.51 | -1.39 | 1.23 |
| **Functional Impairment**  (WHODAS 2.0) | 234 | 1.52 | 0.63 | 0.25 | 3.50 |
| **Personality Dysfunction**  (LPFS-BF 2.0) | 235 | 1.57 | 0.64 | 0.15 | 3.00 |
| **PID-5 Domains** |  |  |  |  |  |
| Negative Affectivity | 197 | 1.48 | 0.61 | 0.13 | 2.72 |
| Detachment | 197 | 1.17 | 0.51 | 0.08 | 2.52 |
| Antagonism | 197 | 0.78 | 0.53 | 0.00 | 2.74 |
| Disinhibition | 197 | 1.65 | 0.39 | 0.94 | 2.90 |
| Psychoticism | 197 | 0.91 | 0.52 | 0.03 | 2.42 |
| **PID-5 Facets** |  |  |  |  |  |
| Anxiousness | 196 | 1.54 | 0.73 | 0.00 | 2.89 |
| Callousness | 197 | 0.54 | 0.43 | 0.00 | 2.71 |
| Depressivity | 197 | 1.27 | 0.75 | 0.00 | 3.00 |
| Eccentricity | 197 | 1.34 | 0.78 | 0.00 | 3.00 |
| Emotional Lability | 197 | 1.81 | 0.74 | 0.00 | 3.00 |
| Hostility | 197 | 1.45 | 0.66 | 0.00 | 2.90 |
| Impulsivity | 197 | 1.79 | 0.55 | 0.17 | 2.83 |
| Perceptual Dysregulation | 197 | 0.84 | 0.51 | 0.00 | 2.58 |
| Restricted Affectivity | 197 | 0.99 | 0.65 | 0.00 | 3.00 |
| Risk Taking | 197 | 1.26 | 0.47 | 0.08 | 2.93 |
| Separation Insecurity | 197 | 1.10 | 0.78 | 0.00 | 3.00 |
| Submissiveness | 197 | 1.56 | 0.72 | 0.00 | 3.00 |
| Suspiciousness | 197 | 1.26 | 0.49 | 0.00 | 2.71 |
| Unusual Beliefs | 196 | 0.54 | 0.52 | 0.00 | 2.43 |
| Withdrawal | 197 | 1.24 | 0.76 | 0.00 | 2.90 |

*Note*. N = sample size; M = mean; SD = standard deviation. ADHD Symptoms measured with Current Symptom Scale (CSS) and Adult ADHD Self-Report Scale (ASRS v.1.1); Functional Impairment assessed using the World Health Organization Disability Assessment Schedule (WHODAS 2.0); Personality Dysfunction measured with the Level of Personality Functioning Scale–Brief Form (LPFS-BF 2.0). Domains and Facets assessed using the Personality Inventory for DSM-5 (PID-5). ADHD symptoms were converted to z-scores; all other scores are raw scores.
