# Supplementary material for: Personality Pathology and Functional Outcomes During Pharmacological Treatment of Adult ADHD
Source: Personal Ment Health. 2026 Mar 29;20(2):e70071. doi: 10.1002/pmh.70071 (PMC13033909; doi:10.1002/pmh.70071)
Supplement: Supplementary file 2 — Table S2: Follow‐up (time span) and number of measuring points per individual. [file PMH-20-0-s003.docx]

**Supplementary Table S2**

Follow-up (time-span) and number of measuring points per individual

|  | Median (IQR) | Range | N |
| --- | --- | --- | --- |
| Follow-up (days) | 735 [383–1127] | 7–2318 | 246 |
| Number of measuring points | 2 [2–3] | 2–7 | 246 |
