# Supplementary material for: Personality Pathology and Functional Outcomes During Pharmacological Treatment of Adult ADHD
Source: Personal Ment Health. 2026 Mar 29;20(2):e70071. doi: 10.1002/pmh.70071 (PMC13033909; doi:10.1002/pmh.70071)
Supplement: Supplementary file 3 — Supporting Information S3: Descriptives of data extraction of medication. [file PMH-20-0-s006.docx]

**Supplementary Material S3**

**Descriptives of Data Extraction of Medication**

Medication records allowed up to three concurrent prescriptions per participant. At each assessment, the prescription module specified the current agent and dose, along with any changes within the preceding 30 days; if a participant had recently increased Elvanse from 35 mg to 70 mg, the earlier dose was recorded for that timepoint. Based on available follow-up data, 46 participants (19%) discontinued medication during the study, and 4 (2%) never initiated treatment despite inclusion. Prescribed agents included both stimulant and non-stimulant medications, most frequently Elvanse (n = 126), Concerta (n = 57), Attentin (n = 29), Ritalin (n = 20), Intuniv (n = 11), Medikinet (n = 6), Atomoxetine (n = 6), Methylphenidate (n = 6), Equasym (n = 2), and Strattera (n = 1).
