# Supplementary material for: Personality Pathology and Functional Outcomes During Pharmacological Treatment of Adult ADHD
Source: Personal Ment Health. 2026 Mar 29;20(2):e70071. doi: 10.1002/pmh.70071 (PMC13033909; doi:10.1002/pmh.70071)
Supplement: Supplementary file 4 — Supporting Information S4: Conversion and rationale amphetamine equivalence. [file PMH-20-0-s001.docx]

**Supplementary Material S4**

**Conversion and rationale amphetamine equivalence**

To facilitate comparisons across stimulant formulations, doses were standardized to amphetamine-equivalents (mg/day), calculated as:

- Methylphenidate (Concerta, Ritalin, Medikinet, Methylphenidate, Equasym) dose ÷ 2
- Lisdexamphetamine (Elvanse) dose ÷ 3.3
- Dexamphetamine (Attentin) dose ÷ 1

Non-stimulants (Intuniv, Atomoxetine, Strattera) were excluded from these calculations. Mean stimulant doses were computed at each study timepoint. At baseline, the mean dose was 21.5 mg (range: 5.0–63.0 mg). Dose adjustments over time were examined by comparing each participant’s initial and final amphetamine-equivalent doses. Among 206 participants with complete data:

- 21 (10.2%) increased their dose
- 17 (8.3%) decreased their dose
- 168 (81.6%) maintained a stable dose

Although stimulant dosages were standardized to amphetamine-equivalents to allow cross-medication comparisons, these values reflect *point estimates* at each self-report assessment. Detailed titration data and dosage changes between measurement points were not available. As a result, accumulated *time in treatment* was considered a more reliable proxy for medication exposure than dosage per se, as it better captures the longitudinal impact of pharmacological treatment despite within-person variability in dosing. Dosage values are therefore interpreted cautiously and primarily as supplementary to treatment duration.
