# Supplementary material for: Personality Pathology and Functional Outcomes During Pharmacological Treatment of Adult ADHD
Source: Personal Ment Health. 2026 Mar 29;20(2):e70071. doi: 10.1002/pmh.70071 (PMC13033909; doi:10.1002/pmh.70071)
Supplement: Supplementary file 5 — Table S5: Internal consistency of the PID‐5 domains and facets. Notes: Cronbach's α and McDonald's ω are measures of internal consistency reliability. Values ≥ 0.70 are generally considered acceptable, though interpretation may vary depending on construct complexity. Mean (SD) values represent the average item scores for each domain or facet of the Personality Inventory for DSM‐5 (PID‐5). Higher scores indicate greater expression of the respective trait. Domain‐level indices (in italics) reflect aggregated facet scores. [file PMH-20-0-s004.docx]

**Supplementary Table S5. Internal consistency of the PID-5 domains and facets**

| **Domains and Facets** | **Cronbach’s α** | **McDonald’s ω** | **Mean (SD)** |
| --- | --- | --- | --- |
| Negative Affectivity | 0.910 | 0.912 | 1.45 (1.04) |
| Detachment | 0.912 | 0.917 | 1.20 (1.01) |
| Antagonism | 0.913 | 0.920 | 0.78 (0.88) |
| Disinhibition | 0.851 | 0.858 | 1.68 (0.87) |
| Psychoticism | 0.939 | 0.939 | 0.93 (0.90) |

| Anhedonia | 0.586 | 0.605 | 1.33 (0.88) |
| --- | --- | --- | --- |
| Anxiousness | 0.659 | 0.690 | 1.43 (1.01) |
| Attention Seeking | 0.634 | 0.676 | 1.15 (1.01) |
| Callousness | 0.769 | 0.802 | 0.82 (0.83) |
| Deceitfulness | 0.778 | 0.797 | 1.10 (0.93) |
| Depressivity | 0.799 | 0.802 | 1.31 (0.94) |
| Distractibility | 0.615 | 0.633 | 1.44 (0.85) |
| Eccentricity | 0.878 | 0.884 | 1.17 (0.98) |
| Emotional Lability | 0.558 | 0.577 | 1.50 (0.96) |
| Grandiosity | 0.666 | 0.687 | 0.91 (0.96) |
| Hostility | 0.741 | 0.750 | 1.32 (1.04) |
| Impulsivity | 0.644 | 0.698 | 1.66 (0.92) |
| Intimacy Avoidance | 0.364 | 0.398 | 1.07 (0.96) |
| Irresponsibility | 0.555 | 0.602 | 1.47 (0.86) |
| Manipulativeness | 0.634 | 0.641 | 0.81 (0.89) |
| Perceptual Dysregulation | 0.733 | 0.746 | 0.94 (0.90) |
| Perseveration | 0.634 | 0.652 | 1.16 (0.96) |
| Restricted Affectivity | 0.636 | 0.669 | 1.01 (0.92) |
| Rigid Perfectionism | 0.593 | 0.602 | 1.14 (0.95) |
| Risk Taking | 0.762 | 0.787 | 1.32 (1.03) |
| Separation Insecurity | 0.746 | 0.759 | 1.14 (1.03) |
| Submissiveness | 0.566 | 0.634 | 1.30 (0.92) |
| Suspiciousness | 0.368 | 0.392 | 1.50 (0.87) |
| Unusual Beliefs | 0.428 | 0.467 | 1.00 (0.91) |
| Withdrawal | 0.736 | 0.768 | 1.26 (1.03) |

Notes: Cronbach’s α and McDonald’s ω are measures of internal consistency reliability. Values ≥ 0.70 are generally considered acceptable, though interpretation may vary depending on construct complexity. Mean (SD) values represent the average item scores for each domain or facet of the Personality Inventory for DSM-5 (PID-5). Higher scores indicate greater expression of the respective trait. Domain-level indices (in italics) reflect aggregated facet scores.
