# Supplementary material for: Personality Pathology and Functional Outcomes During Pharmacological Treatment of Adult ADHD
Source: Personal Ment Health. 2026 Mar 29;20(2):e70071. doi: 10.1002/pmh.70071 (PMC13033909; doi:10.1002/pmh.70071)
Supplement: Supplementary file 6 — Table S6: Sex differences in personality pathology, functional impairment, and ADHD symptoms (independent‐sample t‐tests). Note. N = 246. Independent‐sample t‐tests compare male and female participants. p < 0.05*, *p < 0.01, **p < 0.001. [file PMH-20-0-s002.docx]

**Supplementary Table S6**

Sex Differences in Personality Pathology, Functional Impairment, and ADHD Symptoms
*(Independent samples t-tests)*

|  | *t* | *df* | *p* |  |
| --- | --- | --- | --- | --- |
| General Personality Dysfunction (LPFS-BF 2.0) | −2.11 | 230 | .036* |  |
| Negative Affectivity | −4.58 | 193 | < .001*** |  |
| Detachment | 0.31 | 193 | .759 |  |
| Antagonism | 1.79 | 193 | .074 |  |
| Disinhibition | 0.49 | 193 | .624 |  |
| Psychoticism | 1.78 | 193 | .076 |  |
| Functional Impairment (WHODAS 2.0) | −2.41 | 228 | .017* |  |
| ADHD Symptoms (ASRS/CSS z-mean) | −0.36 | 225 | .717 |  |

*Note.* N = 246. Independent samples t-tests compare male and female participants. *p* < .05*, **p* < .01, ***p* < .001.
