# Supplementary material for: Personality Pathology and Functional Outcomes During Pharmacological Treatment of Adult ADHD
Source: Personal Ment Health. 2026 Mar 29;20(2):e70071. doi: 10.1002/pmh.70071 (PMC13033909; doi:10.1002/pmh.70071)
Supplement: Supplementary file 7 — Table S7: Correlations between age and study variables (Pearson's r). Note. N = 246. LPFS‐BF 2.0 = Level of Personality Functioning Scale—Brief Form 2.0; PID‐5 = Personality Inventory for DSM‐5; WHODAS 2.0 = World Health Organization Disability Assessment Schedule (12‐item version); ASRS = Adult ADHD Self‐Report Scale v1.1; CSS = Barkley ADHD Current Symptoms Scale. p < 0.05*, *p < 0.01, **p < 0.001. [file PMH-20-0-s005.docx]

**Supplementary Table S7. Correlations Between Age and Study Variables** *(Pearson’s r)*

|  | **1.** | **2.** | **3.** | **4.** | **5.** | **6.** | **7.** | **8.** | **9.** |
| --- | --- | --- | --- | --- | --- | --- | --- | --- | --- |
| 1. Age | — |  |  |  |  |  |  |  |  |
| 2. Functional Impairment (WHODAS 2.0) | −.15* | — |  |  |  |  |  |  |  |
| 3. Personality Dysfunction (LPFS-BF 2.0) | −.29*** | .55*** | — |  |  |  |  |  |  |
| 4. Negative Affectivity (PID-5) | −.29*** | .45*** | .62*** | — |  |  |  |  |  |
| 5. Detachment (PID-5) | −.05 | .46*** | .55*** | .35*** | — |  |  |  |  |
| 6. Antagonism (PID-5) | −.07 | .12 | .38*** | .24*** | .16* | — |  |  |  |
| 7. Disinhibition (PID-5) | −.21** | .33*** | .43*** | .34*** | .22** | .44*** | — |  |  |
| 8. Psychoticism (PID-5) | −.18* | .40*** | .54*** | .46*** | .40*** | .45*** | .48*** | — |  |
| 9. ADHD Symptoms (ASRS v1.1/CSS) | .03 | .09 | .17* | .23** | .03 | .22** | .39*** | .21** | — |

*Note****.*** N = 246. LPFS-BF 2.0 = Level of Personality Functioning Scale – Brief Form 2.0; PID-5 = Personality Inventory for DSM-5; WHODAS 2.0 = World Health Organization Disability Assessment Schedule (12-item version); ASRS = Adult ADHD Self-Report Scale v1.1; CSS = Barkley ADHD Current Symptoms Scale.
*p* < .05*, **p* < .01, ***p* < .001.
